# Effects of seasonality and developed land cover on *Culex* mosquito abundance and microbiome diversity

**DOI:** 10.3389/fmicb.2024.1332970

**Published:** 2024-02-08

**Authors:** Jiayue Yan, Kirk Green, Kylee Noel, Chang-Hyun Kim, Chris M. Stone

**Affiliations:** Illinois Natural History Survey, University of Illinois at Urbana-Champaign, Champaign, IL, United States

**Keywords:** *Culex pipiens*, urbanization, bacterial diversity, symbiotic microbiota, vector ecology

## Abstract

The vectorial capacity of mosquitoes, which influences the dynamics of vector-borne disease transmission, is intricately linked to mosquito abundance and the composition and diversity of their associated microbiomes. However, the influence of environmental factors on mosquito populations and microbiome diversity remains underexplored. Here we examined the effects of seasonality and developed land cover on *Culex* mosquito abundance and bacterial diversity. Biweekly field sampling of female *Culex* mosquitoes was conducted using gravid and CDC light traps, spanning summer to autumn across varying developed land cover levels in two urban areas in Central Illinois. Mosquito abundance was assessed by the number of mosquitoes captured per trap night and compared across seasons and developed levels. The mean mosquito abundance for gravid and light traps was 12.96 ± 2.15 and 7.67 ± 1.44, respectively. Notably, higher levels of developed land cover exhibited higher *Culex* abundance than the low level for light traps, but no significant difference was found between summer and early autumn. In gravid traps, no significant differences were detected across seasons or developed levels. Microbial analysis of the mosquito microbiome revealed that Proteobacteria and *Wolbachia*, with a mean relative abundance of 80.77 and 52.66% respectively, were identified as the most dominant bacterial phylum and genus. Their relative abundance remained consistent across seasons and developed land cover levels, with negligible variations. Alpha diversity, as measured by observed species, Chao1, Shannon, and Simpson, showed slightly higher values in early-autumn compared to late-summer. A notable pattern of bacterial diversity, as indicated by all four diversity indexes, is evident across varying levels of land development. Significantly, high or intermediate developed levels consistently showed reduced alpha diversity when compared to the lower level. This underscores the pronounced impact of anthropogenic ecological disturbances in shaping mosquito microbiomes. Beta diversity analysis revealed no significant dissimilarities in bacterial community composition across seasons and developed levels, although some separation was noted among different levels of developed land cover. These findings highlight the significant role of environmental factors in shaping mosquito abundance and their associated microbiomes, with potential implications for the vectorial capacity in the transmission of vector-borne diseases.

## Introduction

Mosquito-borne diseases pose a significant threat to global public health, and continue to be among the most important causes of morbidity and mortality due to infectious diseases worldwide (Vos et al., 2020). The transmission dynamics and epidemiology of vector-borne diseases are tied to spatial and temporal variation among multiple environmental factors, including seasonal changes in temperature and precipitation, and spatial variation in land cover, abundance and composition of host communities, and the composition of vector communities. The vector component in these systems is often summarized by focusing on their vectorial capacity, which isolates the vector-related components of the basic reproduction number of the disease system in question. Traits that are often focused on include vector abundance, longevity, biting behavior, and aspects related to the development of specific viruses in mosquito species (incubation periods and competence). Because these can vary drastically within the same species, understanding the environmental factors that shape these components of vectorial capacity is critical to predicting variation in transmission intensity.

In the United States, West Nile virus has emerged over the past two decades as the most common mosquito-borne virus, for which *Culex* species are the primary vectors (Kilpatrick et al., 2008; Kilpatrick, 2011), leading to hundreds of human cases yearly across much of the country (e.g., Soto et al., 2022). West Nile virus is maintained in a cycle between a variety of primarily avian species and ornithophilic vectors such as *Culex pipiens* and *Cx. restuans* in the north-eastern parts of the US.

Land cover associated with urbanized environments has been linked to WNV transmission and *Culex* spp. abundance. In some cases highlighting negative correlations between highly urbanized environments and mosquito abundance (Moise et al., 2018), whereas in other locations positive correlations have been noted (Deichmeister and Telang, 2011; Wilke et al., 2021). A variety of factors is likely involved, including the ability of *Culex* spp. to exploit types of urban infrastructure such as sewer overflow systems (Deichmeister and Telang, 2011), and the extent to which different host species are fed on in different environments (González et al., 2020).

In addition to vector traits such as abundance, longevity, and biting rates, the microbiome harbored by mosquitoes has emerged as a critical factor influencing their vectorial capacity, with a particular emphasis on bacterial communities. The mosquito microbiome consists of various microorganisms inhabiting the mosquito’s body, including bacteria, viruses, and fungi. Among these, bacterial communities are particularly thought to play a unique and critical role in the mosquito’s ability to transmit pathogens (Bian et al., 2010; Cirimotich et al., 2011; Ricci et al., 2012). Bacteria can interact with the mosquito’s physiology, impacting their immune response, nutrition, and even the production of molecules that affect pathogen transmission (Cirimotich et al., 2011; Gendrin et al., 2015). Bacteria residing in the mosquito gut, such as the genus *Wolbachia*, can also influence the mosquito’s susceptibility to infection and play a pivotal role in limiting pathogen replication (Bian et al., 2010, 2013; Dodson et al., 2014; Zélé et al., 2014).

*Wolbachia* are a genus of obligate endosymbiotic bacteria that are transmitted maternally, that is widespread and common among arthropods. While for a number of mosquito-virus systems *Wolbachia* infection has been shown to limit viral replication, the impact of this endosymbiont on *Culex* species and their interactions with WNV and other pathogens are more equivocal. For instance, *Wolbachia* enhanced the prevalence of salivary gland-stage infections for the avian malaria parasite *Plasmodium relictum* (Zélé et al., 2014). In *Cx. tarsalis*, an important vector in the western parts of the United States which naturally does not harbor *Wolbachia*, infection with *Wolbachia* was shown to enhance the infection rate with WNV, while Rel1, an activator of the Toll immune pathway was down regulated (Dodson et al., 2014). Inhibition of WNV by *Wolbachia* was found for a lab colony of *Cx quinquefasciatus* (Micieli and Glaser, 2014). It has also been suggested however that *Wolbachia* densities in wild *Cx. pipiens* populations may be too low to significantly affect their competence for WNV, or that effects may be population or environment-specific (Micieli and Glaser, 2014). For instance, larval competition has been shown to modulate the effect of *Wolbachia* on WNV competence in *Cx. quinquefasciatus* (Alomar et al., 2023).

The number of studies that has explored interactions between the whole microbiome of *Culex* mosquitoes and their interactions with WNV have been more limited, but suggest that bacteria beyond *Wolbachia* may also be relevant, including those in the genus *Serratia* and *Enterobacter* (Zink et al., 2015; Alomar et al., 2023).

Numerous ecological factors can affect mosquito abundance, life history traits, and bacterial microbiome composition, ultimately shaping their vectorial capacity. Some of these factors include temperature, precipitation, habitat type, host availability, and land cover. Here, we focus on the specific influence of seasonality and developed land cover on *Culex pipiens* / *restuans* mosquito abundance and bacterial microbiome composition, due to the inherent importance of seasonality and associations with urbanized areas.

Seasonality, marked by fluctuations in temperature and precipitation, has a profound impact on mosquito life cycles and bacterial microbiome composition (Krajacich et al., 2018). In cooler seasons, mosquito development and reproduction may slow down, potentially altering the structure of bacterial communities. Conversely, warmer, wetter seasons can provide conducive conditions for certain bacteria to proliferate and may also impact the composition of these microbial communities (Onyango et al., 2020; Lv et al., 2023). Seasonal shifts were for instance observed in microbiome composition of *Culex pipiens*/*restuans* in Ontario, Canada, and for *Wolbachia* in particular the changes in abundance were found to correlate negatively with temperature, as well as with WNV prevalence in tested mosquito pools (Novakova et al., 2017).

Less is known however regarding the way urbanized or developed land, or inversely, the amount of natural land, green areas, or rural environments, shape *Culex* spp. microbiota. Wooded land cover, for instance, can provide a refuge for mosquitoes, harbor different host communities, and provide more natural larval development sites for mosquitoes, and all these factors could influence their acquisition and maintenance of their associated bacterial communities. The shading and high humidity in wooded areas create microclimates favorable for the development of different bacterial species, potentially contributing to acquisition of different microbes. Moreover, wooded areas often host diverse flora and fauna, influencing mosquito feeding patterns and thereby potentially exposing them to a broader range of bacteria (Muturi et al., 2019). In more urbanized areas, we would expect larval development to rely more strongly on urban infrastructure such as catch basins or container habitats, and reliance on human-associated rather than natural larval habitats can affect microbiome composition (Juma et al., 2021).

The Intermediate Disturbance Hypothesis (IDH) posits that moderate levels of disturbance in ecosystems can promote species diversity (Fox, 1979; Sheil and Burslem, 2013). Here, we explore this in the context of mosquito microbiomes by classifying developed land cover into three levels, including an intermediate level that represents a balance between relatively undisturbed wooded areas and highly developed areas. This intermediate level of disturbance may have a unique influence on *Culex* mosquito populations and their bacterial microbiome, promoting diversity in bacterial communities and potentially enhancing the mosquito’s vectorial capacity in unforeseen ways. Despite the recognized importance of ecological factors in shaping mosquito vectorial capacity, there are significant knowledge gaps in understanding how seasonality and wooded land cover interact to influence *Culex* mosquito populations and their bacterial microbiome. This study aims to bridge these gaps and shed light on the nuanced relationships between environmental variables, mosquito abundance, and bacterial microbiome composition in *Culex* mosquitoes. By doing so, we hope to contribute to the development of more effective strategies for mosquito-borne disease prevention and control.

## Methods

### Study sites and mosquito sampling

**Figure 1:**
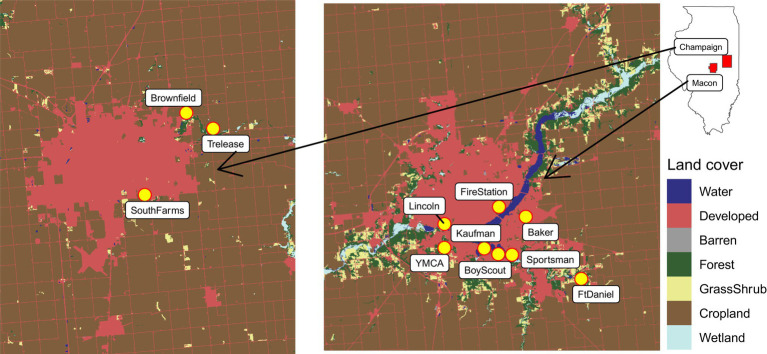
Overview of the sampling sites (in yellow) and land cover classes of the surrounding areas in Central Illinois. Sampling sites with high developed levels: Baker, FireStation, and Lincoln. Sampling sites with intermediate developed levels: BoyScout, Kaufman, SouthFarms, Sportsman, and YMCA. Sampling sites with low developed levels: Brownfield and FtDaniel.

Mosquito sampling was conducted biweekly from June 22 to October 9 in 2018. Northern hemisphere summer solstice (June 21) and autumn equinox (September 22) was used as the cutoff date for classifying our sampling seasons into summer and autumn, respectively. Mosquito sampling was carried out at eight sites in Macon County with the following descriptive names and coordinates: BoyScout (39.80516667, −88.91508333), Kaufman (39.81056667, −88.92943333), YMCA (39.81343333, −88.97131667), Lincoln (39.832407, −88.969417), Baker (39.83335, −88.8833), FireStation (39.842845, −88.910617), Sportsman (39.80387, −88.900742), and FtDaniel (39.78051667, −88.82981667) and three sites in Champaign County: SouthFarms (40.085, −88.214722), Trelease (40.13083333, −88.14083333), and Brownfield (40.144444, −88.166389). These sampling sites were selected from a varying range of developed land cover proportions from 0.05 to 0.91, and further classified as low (below 25th quantile), intermediate (between 25 and 75th quantiles) and high (above 75th quantile) levels (Hereafter ‘developed level’) according to the distribution of the values. Sampling sites with low developed levels include Brownfield and FtDaniel. Sampling sites with intermediate developed levels include BoyScout, Kaufman, SouthFarms, Sportsman, and YMCA. Sampling sites with high developed levels include Baker, FireStation, and Lincoln. Developed land cover data were originally extracted from National Land Cover Database (NLCD) by focusing on a circle with 1 km radius centered on our sampling sites during 2018. Location and land cover of the sampling sites can be seen in Figure 1.

The areas in which we sampled ranged from small fragments of wooded land surrounded by residential or developed land to larger forested areas on the edge of the urban environment. To avoid species-specific sampling biases (Reiskind et al., 2017), the edge or ecotone of the wooded areas (i.e., exterior location), as well as approximately 20 m within the wooded areas (i.e., interior location) were chosen as sampling locations for each site. At each sampling location, both CO_2_-baited CDC light traps (hereafter ‘light trap’) and gravid traps were used. The count of specimens captured at both exterior and interior locations from a trap type was combined for each sampling site. Each sampling event was carried out from approximately noon to noon of the next day. Samples were transported back to the lab in coolers filled with dry ice and stored at-80°C. Identifications were done on chill tables using taxonomic keys (Ross and Horsfall, 1965; Darsie and Ward, 2005). Data on the overall mosquito species assemblage will be described elsewhere. The focus of this study is on *Culex pipiens*, *Culex restuans*, and the unclassified species either *Cx. pipiens* or *Cx. restuans* (hereafter ‘*Cx*. spp.’).

### DNA extraction and Illumina MiSeq sequencing

A total of 227 adult female *Culex* mosquitoes randomly selected to represent various levels of season and developed land cover were processed individually for microbial analysis. Genomic DNA was isolated using DNeasy Blood & Tissue kit (Qiagen, Valencia, CA, USA) with a few modifications. Prior to DNA isolation, each individual mosquito was rinsed once in 70% ethanol for 10 min and 5 times in sterile Dulbecco’s phosphate-buffered saline (DPBS) solution (Thermo Fisher Scientific) for 10 s and placed in a 2-ml microcentrifuge tube containing 3 stainless steel beads (3.2 mm diameter, BioSpec Products, Bartlesville, OH, USA) and lysis buffer consisted with 50 μL of buffer ATL (Qiagen) and 20 μL of proteinase K (Qiagen). After homogenizing mosquito body using TissueLyser II (Qiagen) at 30 beats/s for 5 min, DNA isolation was performed following manufacturer’s protocol. The volume of buffer AE (Qiagen) at elution step was 100 μL.

The resulting DNA isolate was utilized to build a microbiome library and was sequenced at the W. M. Keck Center for Comparative and Functional Genomics at the University of Illinois at Urbana-Champaign. For bacterial characterization, we targeted the V4 hypervariable region of the 16S rRNA gene using the following primer set: forward 5’-GTGYCAGCMGCCGCGGTAA-3′ and reverse 5’-GGACTACNVGGGTWTCTAAT-3′ (Apprill et al., 2015; Parada et al., 2016). Briefly, DNA of each mosquito sample was amplified using the primer set on the Fluidigm microfluidics quantitative PCR platform with appropriate linkers and sample barcodes. The final Fluidigm libraries were pooled in equimolar ratio and sequenced by 2 × 300 nt paired-end sequencing on the Illumina MiSeq Bulk v3 system.

### Sequence processing

Primer sequences were removed from the data using cutadapt version 4.1 (Martin, 2011), with the default parameters, which included a maximum error rate of 10% and the utilization of the-g flag to remove bases located upstream of the primers. Subsequent amplicon processing was conducted in R software version 4.3.1 (R Development Core Team, 2022) with the DADA2 package version 1.28.0 (Callahan et al., 2016). The quality of the reads was assessed through the plotQualityProfile function. Filtering and trimming of low-quality reads involved adjustments to parameters such as truncLen (250, 210), minLen (20), truncQ (2), and maxEE (2, 2) in the filterAndTrim function, taking into account the overall sequence quality. Forward and reverse reads were merged using the mergePairs function, adhering to default parameters with a minimum overlap of 12 and no allowed mismatches. Chimeric sequences were removed using removeBimeraDenovo with default settings. Taxonomic classification of amplicon sequence variants (ASVs) was carried out using the assignTaxonomy function from dada2, referencing the SILVA database (Callahan, 2018) version 132.^1^ Bootstrap values were not subjected to any thresholding (minBoot = 0).

### Statistical analyses

Total *Culex pipiens/restuans* mosquito abundance at each sampling site was quantified by summing the number of female *Cx. pipiens*, *Cx. restuans*, and *Cx*. spp. collected from both types of traps throughout the sampling seasons. To mitigate any potential sampling effort disparities among different seasons and developed levels, the count of female *Culex* mosquitoes captured by each individual trap during each night (count per trap night) served as a proxy for mosquito abundance per sampling. This observation was adopted to examine whether seasonality (summer vs. autumn) or developed levels (high vs. low, intermediate vs. low) had any significant effects using Wilcoxon rank sum. By exploring the impact for CDC light trap and gravid traps this allows for a distinction between host-seeking and oviposition-site seeking female mosquitoes, capturing subpopulations differing in their age and physiological profile.

Bacterial ASV diversity analyses were performed using the phyloseq R package (McMurdie and Holmes, 2013). Before the analyses, 37 eukaryotic ASVs were removed; 15 ASVs belonging to Kingdom Bacteria but lacking taxonomic assignments at lower classifications were manually blasted and their taxonomic names based on a 97% Identity threshold and 100% coverage were added. Reads of samples were normalized to median sequencing depth following DESeqVS method (McMurdie and Holmes, 2014; Vaulot et al., 2022; The Phyloseq Tutorial, 2023). The relative abundance of the most dominant phylum and genus was compared between trap types, sampling seasons, and developed levels, respectively, using Wilcoxon rank sum. Alpha diversity metrics, including observed species, Chao1, Shannon, and Simpson diversity index were generated and their means across trap types, seasons and developed levels were compared using Wilcoxon rank sum. To visualize potential structural dissimilarities in bacterial communities, beta diversity analysis was conducted using weighted UniFrac dissimilarity for Principal Coordinates Analysis (PCoA) and Constrained correspondence analysis (CCA) plots. The weighted UniFrac dissimilarity was chosen due to its compatibility with DESeqSV normalization (Weiss et al., 2017). Using the ‘adonis2’ function within the vegan package (Oksanen et al., 2007), a Permutational Analysis of Variance (PERMANOVA) test with 999 permutations was performed to assess if there is significant structural dissimilarity in bacterial communities between traps, seasons, and developed land cover. The results of the above comparisons were plotted in boxplots by the ggpubr package (Kassambara, 2020). All analyses were carried out in R software version 4.3.1 (R Development Core Team, 2022).

## Results

**Figure 2:**
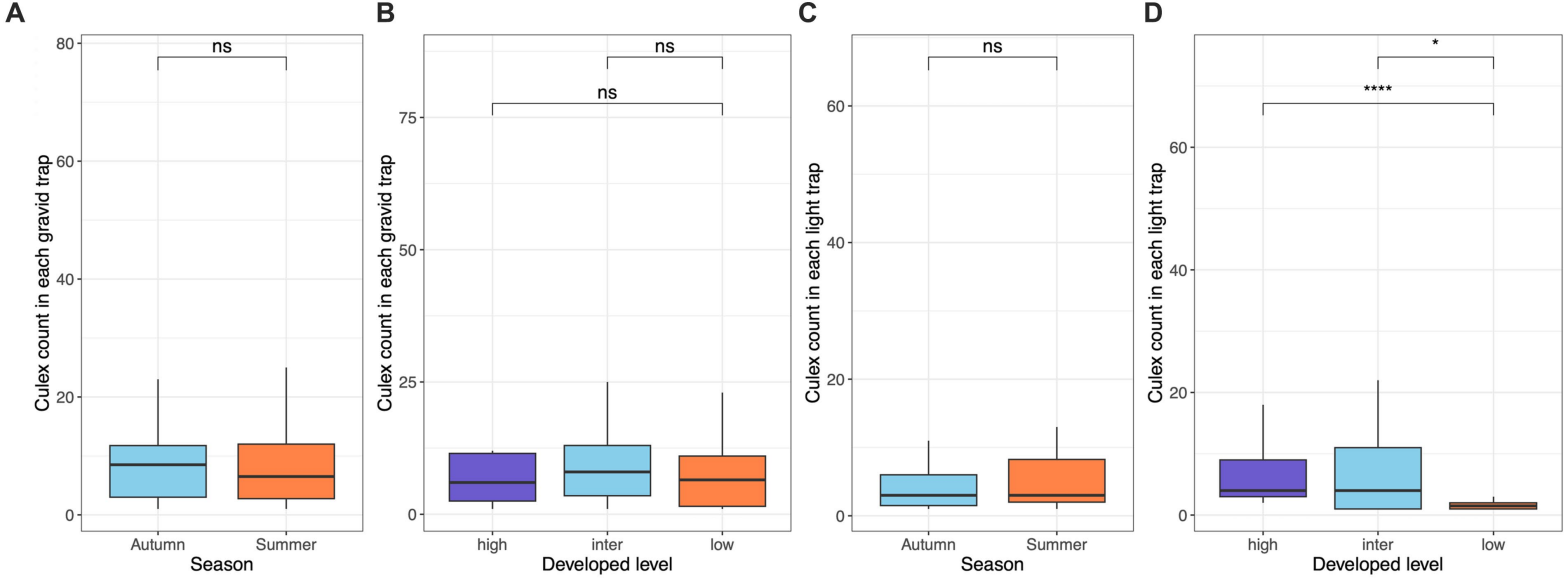
*Culex pipiens/restuans* mosquito abundance in gravid and light traps across levels of developed land use and seasons. The abundance was measured as the count of females captured by each trap each night. Abundance in gravid traps exhibited no difference across seasons **(A)** and developed levels **(B)**. Abundance in light trap exhibited no difference across seasons **(C)**, while there were significant differences both between high and low levels (Wilcoxon test, *p* < 0.001) and between intermediate and low levels (Wilcoxon test, *p* = 0.02) of developed land cover **(D)**.

We collected a total of 829 *Cx. pipiens/restuans* from gravid traps and 560 from light traps over the course of the study. The total mosquito abundance for each sampling site ranged from 27 to 332, with Kaufman displaying the lowest count, while SouthFarms exhibited the highest count. The count of *Culex* mosquitoes captured at each site, categorized by gravid or light traps, is provide in Supplementary Table S1. Mosquito abundance at individual gravid traps ranged from 1 to 74, with a calculated mean ± standard error (SE) of 12.96 ± 2.15, while for light traps, the range was 1 to 64, with a mean ± SE of 7.67 ± 1.44. Notably, mosquito abundance at gravid traps did not exhibit significant differences between autumn and summer seasons (Wilcoxon test, *p* = 0.66; Figure 2A), nor did it vary across low, intermediate, and high levels of developed land cover (Wilcoxon test, both *p* > 0.05, Figure 2B). In the case of light traps, while there was no significant difference between autumn and summer (Wilcoxon test, *p* = 0.81; Figure 2C), noteworthy distinctions emerged: the high and intermediate levels of developed land cover displayed significantly higher mosquito abundance compared to the low level, respectively (Wilcoxon test, *p* < 0.001 & *p* = 0.02, Figure 2D).

When comparing microbiomes of mosquitoes collected in either gravid or light traps, we found that there were no statistically significant differences in the relative abundance of Proteobacteria (Wilcoxon test, *p* = 0.54) or any of the four alpha diversity indexes (Wilcoxon test, all *p* > 0.05) between trap types. However, notable distinctions were observed in the relative abundance of *Wolbachia* (Wilcoxon test, *p* < 0.001) and beta diversity (adonis2 test, *p* < 0.001) between trap types. Because of this variation between trap types, and considering that 184 out of the 227 samples selected for microbial analyses originated from gravid traps, our subsequent microbial analyses exclusively focus on samples collected from gravid traps (*N* = 184). Furthermore, to address the potential impact of unbalanced sampling efforts during different seasons, we categorized our data into three distinct levels: early-to-mid-summer (June 22 to August 31, *N* = 11), late-summer (September 1 to 21, *N* = 91), and early-autumn (September 22 to October 9, *N* = 82). Consequently, our microbial analyses will concentrate solely on samples collected from gravid traps during the late-summer and early-autumn sampling seasons (*N* = 173).

**Figure 3:**
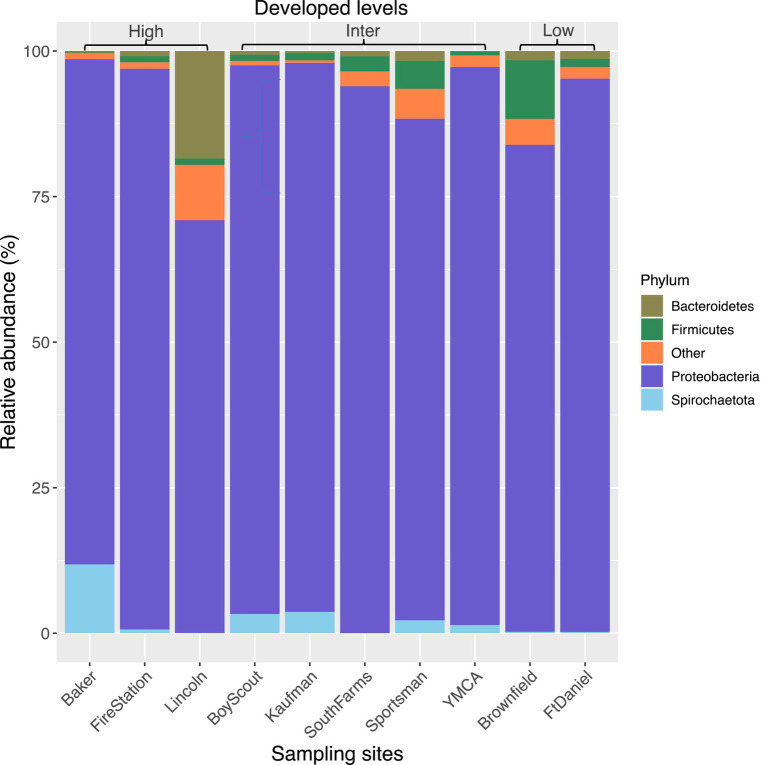
Bacterial composition in mosquitoes collected from gravid traps at 10 sites in central Illinois. Data represent taxonomic classification at phylum level. Phyla with relative sequence abundance <1% of total sequences were pooled together as “Other.” “High,” “Inter,” and “Low” represents high, intermediate, and low level of developed land cover, respectively. Number of mosquitoes analyzed for each site from left to right is 20, 15, 20, 19, 7, 13, 20, 25, 14, and 20.

**Figure 4:**
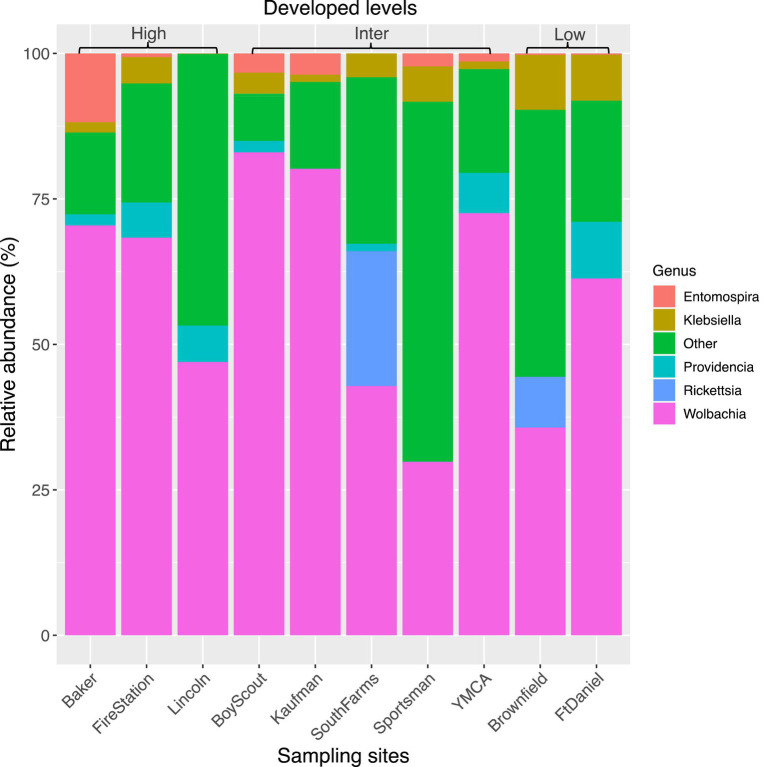
Bacterial composition in mosquitoes collected from gravid traps at 10 sites in central Illinois. Data represent taxonomic classification at genus level. Genera with relative sequence abundance <2% of total sequences were pooled together as “Other.” “High,” “Inter,” and “Low” represents high, intermediate, and low level of developed land cover, respectively. Number of mosquitoes analyzed for each site from left to right is 20, 15, 20, 19, 7, 13, 20, 25, 14, and 20.

**Figure 5:**
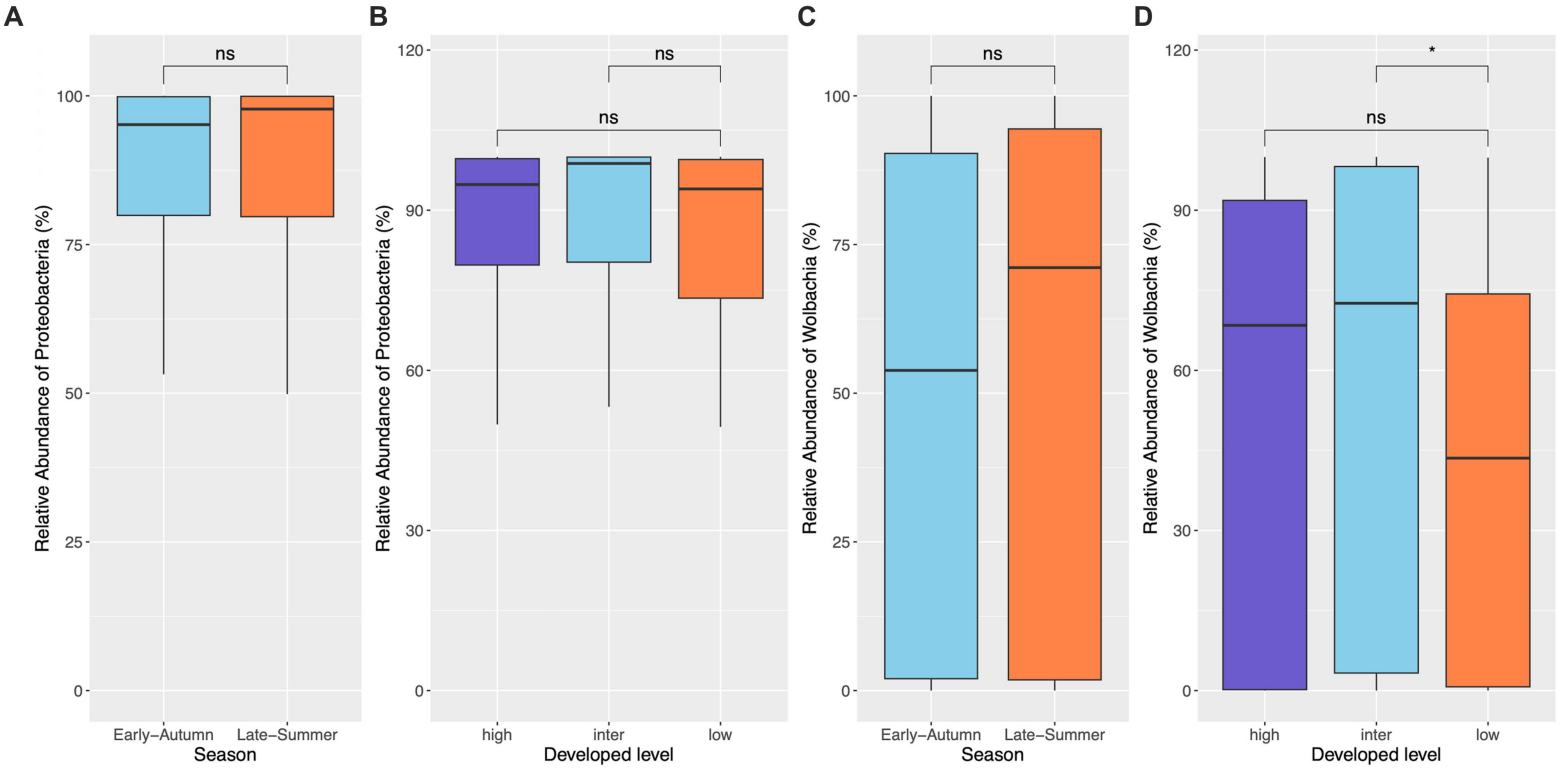
Relative abundance of the dominant phylum (*Proteobacteria*) and genus (*Wolbachia*) in mosquitoes from gravid traps. No difference of the relative abundance of both *Proteobacteria* and *Wolbachia* across seasons **(A,C)** and developed levels **(B,D)** was found, except that the low developed level displayed marginally significantly lower relative abundance of *Wolbachia* than the intermediate level (*p* = 0.048).

**Figure 6:**
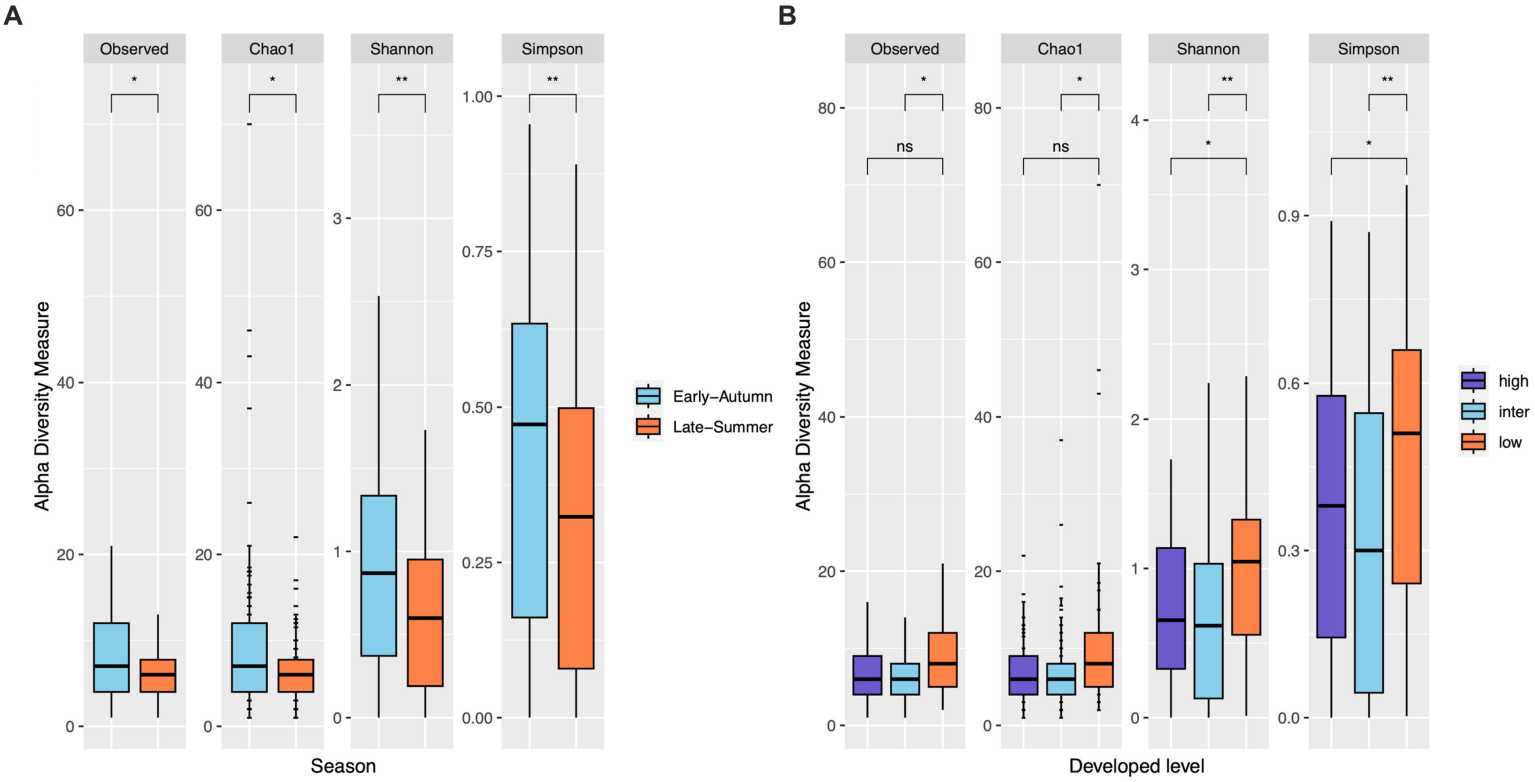
Bacterial ASV diversity within mosquito samples from gravid traps. Alpha diversity is slightly higher in early-autumn than in late-summer for all indexes (Wilcoxon test, both *p* < 0.05 for Observed and Chao1 indexes, both *p* < 0.01 for Shannon and Simpson indexes; **(A)**). Low level of developed land cover exhibited significantly higher alpha diversity than the intermediate level consistently across four indexes (Wilcoxon test, both *p* < 0.05 for Observed and Chao1 indexes, both *p* < 0.01 for Shannon and Simpson indexes; **(B)**), but only the Shannon and Simpson indexes displayed significant difference between low and high levels of developed land cover (Wilcoxon test, both *p* < 0.05).

**Figure 7:**
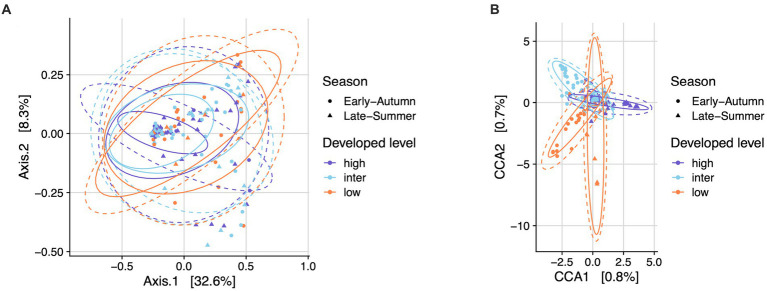
Bacterial community structure in mosquitoes from gravid traps across seasons and developed levels. Plots depicted by principal coordinates analysis (PCoA) **(A)** and canonical correspondence analysis (CCA) **(B)** based on weighted UniFrac distance. Permutational analysis of variance (PERMANOVA) showed no significant dissimilarity across season (adonis2, *p* = 0.13) nor the levels of developed land cover (adonis2, *p* = 0.27).

The top seven bacterial phyla, each with a relative abundance exceeding 1% cumulated from all samples, were Proteobacteria (80.77%), Spirochaetota (4.49%), Firmicutes (4.03%), Actinobacteria (3.90%), Bacteroidetes (2.38%), Cyanobacteria (1.63%), and Epsilonbacteraeota (1.01%). At each sampling site, the four phyla with a mean relative abundance greater than 1% were Proteobacteria, Spirochaetota, Bacteroidetes, and Firmicutes. A detailed depiction of the specific composition of bacterial phyla across sampling sites is illustrated in Figure 3. Furthermore, the top four bacterial genera, each with a relative abundance exceeding 2% cumulated from all samples, were *Wolbachia* (52.66%), *Entomospira* (4.49%), *Klebsiella* (3.45%), and *Providencia* (2.88%). At each sampling site, the five genera with a mean relative abundance greater than 2% were *Wolbachia*, *Entomospira*, *Klebsiella*, *Providencia*, and *Rickettsia*. The specific composition of bacterial genera across sampling sites is detailed in Figure 4. Our analysis revealed no significant variations in the relative abundance of the dominant phylum Proteobacteria across seasons (Wilcoxon test, *p* = 0.76) or developed levels (Wilcoxon test, both *p* > 0.05; Figures 5A,B). Similar findings were observed for the relative abundance of the dominant genus *Wolbachia* (Wilcoxon test, all *p* > 0.05; Figures 5C,D), except there was a marginally significant difference between intermediate and low levels of developed land cover (Wilcoxon test, *p* = 0.048; Figure 5D). Significantly higher alpha diversity of bacterial ASVs measured by all four indexes was detected during early-autumn compared to late-summer (Wilcoxon test, all *p* < 0.05; Figure 6A). Additionally, a significantly higher alpha diversity was observed at the low level of developed land cover compared to the intermediate level consistently for all four indexes (Wilcoxon test, all *p* < 0.05; Figure 6B). The same pattern was observed for the comparison between high and low developed levels, with a significantly higher alpha diversity at the low level than at the high level for both Shannon and Simpson indexes (Wilcoxon test, both *p* = 0.03; Figure 6B). Furthermore, beta diversity analyses, as depicted by both PCoA and CCA, indicated that the dissimilarities in bacterial community composition across seasons or developed levels were not statistically significant (adonis2, for seasons: *p* = 0.13, for developed levels: *p* = 0.27). It is worth noting that the largest proportion of the data variance, as explained by Axis 1 of the PCoA plot, reached 32.6% (Figure 7A). Additionally, the CCA plot displayed some separation among high, intermediate, and low levels of developed land cover (Figure 7B).

## Discussion

Our findings demonstrate that developed land cover can shape mosquito abundance and their microbiome diversity, while seasonality also impacts the diversity of mosquito-associated microbiomes. Particularly, the abundance of *Culex* mosquitoes, measured by the capture per light trap per night, is significantly higher in areas with higher levels of developed land cover compared to those with lower levels. However, the low level of developed land cover exhibits consistently higher microbiome diversity than the higher levels. Mosquitoes collected in early-autumn display higher within-sample bacterial diversity compared to those collected in late-summer. This study contributes to our understanding of the complex interplay between environmental factors, mosquito abundance, and bacterial microbiome composition in *Culex* mosquitoes, shedding light on the implications for vectorial capacity and mosquito-borne disease transmission.

The observed positive correlation between developed levels and *Culex* mosquito abundance, as determined through light trap samples, aligns with previous studies, highlighting the pivotal role of anthropogenic ecological disturbance in shaping mosquito populations (Landau and van Leeuwen, 2012; Thongsripong et al., 2013; Mayi et al., 2020). Developed land cover encompasses a range of characteristics, from open spaces to low-to high-intensity areas featuring a mix of constructed materials and vegetation. These characteristics influence the proportion of impervious surfaces, significantly altering the natural habitats of mosquitoes. Vegetated areas, for instance, due to their shading and high humidity, create microclimates favorable for larval and adult mosquito development, survival and reproduction. In areas with higher levels of developed land cover, characterized by reduced vegetation areas, there may be a decrease in the abundance of mosquito species that rely on natural habitats (Ferraguti et al., 2016). Conversely, for species that have adapted to man-made environments, elevated land development may lead to an increase in mosquito abundance (Wilke et al., 2021). For instance*, Cx. pipiens* has frequently been reported as the most abundant species in urban settings (Ferraguti et al., 2016; Mayi et al., 2020). Additionally, Landau and van Leeuwen (2012) demonstrated a positive association between the presence of a human-adapted mosquito species, *Cx. quinquefasciatus*, and the presence of pavement, a specific type of impervious surface. This further underscores the intricate relationship between land development and mosquito abundance, with implications for public health and vector-borne disease transmission. Curiously, the observed positive correlation between developed levels and the abundance of *Culex* mosquitoes appears to hinge on the sampling traps, as no significant effect was noted in gravid-trap samples. It is possible that this reflects a difference in population structures between developed land categories, with a greater proportion of younger, host-seeking females in more developed areas. Alternatively, it could reflect a difference in capture efficiency in different habitats (e.g., the effectiveness of the light trap could be affected by the amount and density of vegetation or of artificial light at night in the environment). Further research on the age structures of populations in different levels of developed land use would be beneficial.

Surprisingly, our analysis revealed no discernible seasonal impact on *Culex* mosquito abundance when comparing the summer and autumn periods in this study. Seasonal variations in temperature and precipitation play pivotal roles in shaping mosquito development and reproduction rates (Helbing et al., 2015; Soh and Aik, 2021). Prior research has demonstrated that fluctuations in rainfall across seasons can exert a direct influence on both mosquito diversity and population dynamics (Muturi et al., 2006). One potential explanation for the absence of seasonal disparities in our findings is that there was minimal, if any, dramatic change in temperature and precipitation between our sampling periods. In Central Illinois, the mean daily temperature and precipitation changed only modestly, shifting from 23.8°C and 2.96 mm in summer (June 21st to September 21st) to 19.7°C and 1.89 mm in autumn (September 22nd to October 9th) of 2018, according to data from the National Weather Service (2023). It is noteworthy that certain human-adapted mosquito species, such as *Cx. pipiens*, have acclimated to man-made habitats and may exhibit reduced responsiveness to mild seasonal fluctuations in precipitation (Valentine et al., 2020). To better capture seasonal effects, future studies might consider sampling locations with more pronounced temperature and precipitation variations. Alternatively, the mosquito species under examination in our study, including *Cx. pipiens* and *Cx. restuans*, are widespread and tend to exhibit slightly distinct seasonal patterns. *Cx. restuans*, for instance, tends to peak earlier in the summer, while *Cx. pipiens* peaks later. It is conceivable that the chosen sampling periods in our study encompassed both of these distinct peaks, potentially offsetting their seasonal effects.

The identification of Proteobacteria and *Wolbachia* as the most dominant bacterial phyla and genera in our study is consistent with previous research on *Culex* mosquito microbiomes (Novakova et al., 2017; Bergman and Hesson, 2021; Schrieke et al., 2022; Suo et al., 2022). Proteobacteria is a common phylum found in mosquito guts (Chandel et al., 2013; Duguma et al., 2015; Mancini et al., 2018; Suo et al., 2022), and *Wolbachia*, a genus residing in mosquito gut and reproductive tissues, can influence susceptibility to infection and limit pathogen replication (Bian et al., 2010). The presence of these dominant taxa in our study underscores the stability of these associations in *Culex* mosquitoes. Notably, the relative abundance of these phyla and genera exhibited distinct compositional patterns across the various sampling sites (Figures 3, 4). Interestingly, the relative abundance of *Wolbachia* displayed considerable variability, ranging from 30% to as high as 80% from site to site. It’s worth mentioning that while some previous studies have reported *Wolbachia* relative abundances of up to 80–90% in *Cx. pipiens* (Bergman and Hesson, 2021; Schrieke et al., 2022), the relative abundance of *Wolbachia* in our study was approximately 52%. The disparities observed between studies warrant further investigation. To delve deeper into these variations, we assessed whether factors such as seasonality and land development contributed to the differences by comparing the relative abundance of the dominant phylum Proteobacteria and genus *Wolbachia*. Neither of these factors had a significant impact on the abundance of Proteobacteria, but the relative abundance of *Wolbachia* was found to be lower in the low developed level, although this was only significant in comparison to the intermediate level (Figure 5). How does this phenomenon measure up in relation to alterations in the absolute abundance of *Wolbachia*, whether such changes have an impact on WNV transmission, and whether and how other environmental factors besides seasonal changes and land use variation shape the abundance of these dominant bacterial taxa are questions that warrant further research.

Seasonality’s influence on microbial diversity was evident, with early-autumn exhibiting higher alpha diversity than late-summer, as indicated by all four diversity indices (see Figure 6A). Previous studies have also reported significant variations in bacterial diversity across different seasons. For instance, Duguma et al. (2017) demonstrated marked differences in the bacterial communities associated with immature life stages of *Culex* mosquitoes across summer, autumn, and winter seasons. As temperatures increase during warmer months, microbial populations tend to flourish due to heightened metabolic activity. Furthermore, alterations in precipitation patterns throughout the seasons can impact the microbial communities within mosquito breeding sites, subsequently influencing the microbial diversity found in adult mosquitoes (Sandeu et al., 2022). The relatively minor distinction observed between the late-summer and early-autumn seasons in our study may be attributed to the absence of substantial fluctuations in daily temperature and precipitation between our sampling periods. This underscores the importance of selecting distinct sampling seasons based on temperature and precipitation variations to capture any significant seasonal effects accurately.

We documented reduced bacterial diversity in *Culex* mosquitoes in urbanized areas with higher developed levels. Urbanization and developed land cover often leads to habitat alterations, including changes in the availability and quality of mosquito breeding sites. Urban areas may have artificial containers such as stormwater drains or discarded containers that can serve as larval habitats. These artificial breeding sites can be influenced by various urbanization-related factors, including pollutants (Guégan et al., 2018) and the urban heat island effect (Duval et al., 2023). Consequently, they may exhibit distinct microbial communities when compared to their counterparts in natural habitats. In addition, developed land cover is often associated with a less diverse avian fauna. Whether the microbial community composition of *Culex* mosquitoes is affected by such natural variations in host abundance and resulting blood-feeding patterns is in need of further research.

Our beta diversity analyses, as revealed by PCoA and CCA, did not identify significant structural dissimilarities in bacterial community composition between seasons or developed land cover levels. However, the slight separation observed among high, intermediate, and low levels of developed land cover in the CCA plot suggests subtle differences in bacterial community composition, potentially due to variations in habitat and resource availability. Our findings do not align with the intermediate disturbance hypothesis, as the intermediate level did not display significantly higher abundance, diversity, or dissimilarity than other levels when assessing the relative abundance of the most dominant phylum and genus, as well as alpha and beta microbial diversity.

Our study has limitations that should be considered in future research. The study was conducted over a single year from June to early October, and longer-term investigations are necessary to capture the full range of seasonal dynamics and understand how microbial communities respond to longer timeframes. To ensure a balanced sample size between the two sampling seasons, we conducted microbial analyses solely on samples from gravid traps. It is possible that the microbial composition and diversity of mosquitoes captured from other traps may exhibit distinct patterns, which could be investigated in future studies. Future research should also delve deeper into the functional aspects of the microbiome, exploring the specific roles of bacterial taxa in modulating vectorial capacity. Additionally, incorporating other ecological factors, such as larval habitat characteristics, could provide a more comprehensive understanding of mosquito ecology and explain the variation in specific taxa.

Understanding the interactions between environmental variables, mosquito abundance, and bacterial microbiome composition is crucial for the development of targeted strategies for mosquito-borne disease control. By revealing the impact of seasonality and developed land cover on *Culex* mosquitoes and their bacterial communities, our study provides valuable insights for disease prevention efforts. These insights may guide the development of interventions that account for the dynamics of mosquito populations and their microbiomes in specific environmental settings.

## Data availability statement

The original contributions presented in the study are publicly available. This data can be found here: NCBI - PRJNA1061157.

## Author contributions

JY: Conceptualization, Data curation, Formal analysis, Investigation, Methodology, Validation, Visualization, Writing – original draft, Writing – review & editing. KG: Data curation, Formal analysis, Investigation, Writing – review & editing. KN: Conceptualization, Investigation, Writing – review & editing. C-HK: Conceptualization, Data curation, Investigation, Methodology, Project administration, Supervision, Writing – original draft, Writing – review & editing. CS: Conceptualization, Data curation, Formal analysis, Funding acquisition, Investigation, Methodology, Project administration, Supervision, Visualization, Writing – original draft, Writing – review & editing.

## Footnotes

^1^ https://zenodo.org/record/1172783
