# Supplementary material for: Effects of seasonality and developed land cover on Culex mosquito abundance and microbiome diversity
Source: Front Microbiol. 2024 Feb 8;15:1332970. doi: 10.3389/fmicb.2024.1332970 (PMC10885804; doi:10.3389/fmicb.2024.1332970)
Supplement: Supplementary file 1 [file Table_1.DOCX]

|  | **High developed level** | | |  | **Intermediate developed level** | | | | |  | **Low developed level** | | |
| --- | --- | --- | --- | --- | --- | --- | --- | --- | --- | --- | --- | --- | --- |
| **Trap type** | **Baker** | **FireStation** | **Lincoln** |  | **BoyScout** | **Kaufman** | **SouthFarms** | **Sportsman** | **YMCA** |  | **Brownfield** | **FtDaniel** | **Trelease** |
| Gravid trap | 161 | 17 | 65 |  | 64 | 14 | 179 | 29 | 134 |  | 72 | 64 | 30 |
| Light trap | 23 | 94 | 67 |  | 7 | 13 | 153 | 115 | 39 |  | 17 | 3 | 29 |

**Table S1. Number of *Culex* mosquitoes captured from each sampling site by different traps**. The levels of developed land cover for sampling sites were shown at the first row of the table.
